# The burden of care and the understanding of disease in Parkinson’s disease

**DOI:** 10.1371/journal.pone.0217581

**Published:** 2019-05-31

**Authors:** Geum-Bong Lee, Hyunhee Woo, Su-Yoon Lee, Sang-Myung Cheon, Jae Woo Kim

**Affiliations:** 1 Department of Neurology, School of Medicine, Dong-A University, Busan, Korea; 2 Department of Nursing, Dongju College, Busan, Korea; 3 Department of Neurology, Pohang S Hospital, Pohang, Korea; Hospital General Dr. Manuel Gea Gonzalez, MEXICO

## Abstract

**Background and objective:**

Education of disease plays an important role in management of Parkinson's disease (PD). However, little is known about the link between the understanding of disease and the burden of care. This study was carried out to find the correlation between the burden of care and the understanding of disease in caregivers for PD patients.

**Methods:**

Non-demented patients with PD and their caregivers participated in structured interviews. Understanding of patients and caregivers was evaluated through newly-devised questions. The caregiver burden inventory was used to assess the burden of care.

**Results:**

A total of 142 pairs of patients and their caregivers were recruited. A correlation analysis showed that the burden of care was positively associated with low understanding of the disease by the caregiver. Daily care time and female patients were revealed to be independently associated with the burden of care through a multivariate analysis. Further analyses were performed in the caregiver group according to relationship with patients. The spouse group showed an increased burden of care and poor understanding compared to the offspring group. A multivariate analysis revealed that daily care time and understanding were independent predictors for the burden of care in the spouse group. There was no significant association in the offspring group.

**Conclusion:**

The burden of care was associated with higher daily caregiving time and female gender of the patient, and was significantly increased in the spouse of the patient. In the spouse group, better understanding of the caregiver correlated with less burden of care.

## Introduction

Parkinson's disease (PD) is a neurodegenerative disease causing the loss of dopaminergic neurons in the substantia nigra, which chronically progresses [1]. Early PD patients undergoing medical treatment do not have significant difficulty in daily living, but patients with advanced PD experience difficulties in basic daily living activities due to various motor and non-motor symptoms, and the disability in daily living continually increases with progression of the disease [1,2]. Specific motor symptoms of PD, including bradykinesia, tremor, rigidity, and gait disturbance, are readily apparent to patients and caregivers, but their interests in and recognition of non-motor symptoms, including gastrointestinal symptoms, cognitive/emotional disorder, autonomic disturbance, and pain are lower [3]. In particular, the non-motor symptoms of PD have been identified as factors that increase burden of care and lower the quality of life more than the motor symptoms [4,5]. This finding has also been confirmed by a report showing that the non-motor symptoms may be the key factors that reduce the quality of life, even in early stages of the disease [6]. Therefore, proper understanding and recognition of the disease by PD patients and caregivers may be improved by providing more knowledge about the disease itself, and lack of understanding of the disease may have a negative effect on the care of PD patients [7,8].

Many PD patients and caregivers have difficulties in identifying the actual symptoms of PD, want to understand the disease, and feel the need for education on the disease [9]. A study of the Patient Education Program for Parkinson's disease (PEPP), provided to PD patients and caregivers, showed that the PEPP gave psychosocial help both to the patients and caregivers [10]. Educational programs on the disease may help patients and caregivers to understand the disease, reduce the burden of care, and improve the quality of life [11]. Disease education programs for the primary caregivers of patients with dementia, another representative neurodegenerative disease, improved the quality of care by decreasing the burden of care [12].

The PEPP is focused mainly on the psychosocial factors, and little has been studied on the correlation between the understanding of the disease and the burden of care [10]. Despite a report showing that education provided to the caregivers of patients with chronic neurodegenerative diseases, including PD, was helpful for their care, studies on specific and objective education programs that may reduce the caregiver's burden have not been sufficiently conducted [9,13]. Therefore, it is necessary to verify the correlations between the actual burden of care and the degree of understanding of the disease.

The present study was conducted to investigate the correlations between the understanding the disease of the caregivers and the burden of care in PD. For this purpose, the factors impacting the burden of care were analyzed, and questions about PD were devised to investigate the degree of understanding of the disease. In addition, the primary caregivers were classified into groups of spouses and offspring, in order to investigate the differences according to the relationship with the patients.

## Methods

The subjects of the present study had been diagnosed with PD without a cognitive disorder (Korean Mini-mental status examination (K-MMSE) > 24), and were stably undergoing management through an outpatient clinic. When patients visited the hospital with their primary caregiver, the structured interview was conducted after receiving a written informed consent. The burden of care of the primary caregivers was evaluated using the Caregiver Burden Inventory (CBI) [14]. The CBI is comprised of five sub-dimensions of time-dependence, developmental, physical, social and emotional burden. A higher CBI score means a higher caregiver burden. In the present study, the CBI questionnaire was modified by adding questions about economic burden. The questions for economic burden were consisted of three questions, but those were not validated prior to applicate in this study. To identify the factors related to caregiver burden, the demographical and clinical characteristics of the patients were investigated, in addition to the relationship between the patient and the primary caregiver, monthly income of the caregiver, time of care, and cost of care. The level of understanding the disease was evaluated by a test prepared by three experts on the physiopathology/diagnosis, symptoms, and treatment/progress. The test consisted of 20 multiple-choice questions.

Informed consent was obtained from all subjects, and the study was approved by the Institutional Review Board of the Dong-A University Medical Center (IRB No. 14–101).

Patient and caregiver baseline and clinical characteristics were summarized by descriptive statistics for continuous variables, and frequency and percentage for categorical variables. Chi-squared test or Fisher’s exact test was used to compare categorical variables between groups, while an independent t test or Mann-Whitney’s U test was used to compare continuous variables between groups. Shapiro-Wilk’s test was employed for tests of normality. The Pearson’s correlation coefficients were estimated to investigate the association between predictors and the response variable. The effect of independent variables on the response variable was analyzed using a multivariate linear regression, and the statistically significant variables were selected in a stepwise manner with a 0.05 alpha level. All statistical analyses were performed using SPSS 24.0 version (SPSS for Windows 24.0, SPSS, Chicago, IL, USA) and p values less than 0.05 were considered statistically significant. By its nature, this study was explorative and therefore no adjustment for multiple testing was applied.

## Results

### Characteristics of subjects

The number of PD patients and their primary caregivers who participated in the present study was 142 each. The number of male PD patients was greater, the average Hoehn and Yahr stage was 2.48, and the average duration of the disease was 72 months (Table 1). The number of primary caregivers was not significantly different between the male and the female caregivers. The average care duration was 59.4 months, and the average daily care time was 7.6 hours. The average monthly cost for care was 331,000 KRW.

**Table 1:** Patient and caregiver baseline characteristics.

		Statistic	Median (range)
**Patients’ characteristics**		N = 142	
	Male : Female	83 : 59	
	Age (years)	69.63±7.25	72.50 (52.50–77.50)
	Hoehn and Yahr stage	2.48±0.52	2.5 (1.00–4.00)
	Duration of disease (months)	72.00±49.75	54.00 (6.00–180.00)
	Education(years)	9.04±4.30	9.00 (0.00–16.00)
	K-MMSE	26.28±3.45	27.00 (14.00–30.00)
**Caregivers’ characteristics**		N = 142	
	Sex	72 : 70	
	Age (years)	59.42±14.40	62.50 (30.00–77.50)
	Education (years)	9.04±4.30	9.00 (0.00–16.00)
	Income (10^4^won/month)	217.08±131.39	225.00 (50.00–425.00)
	Care duration (months)	59.44±50.93	54.00 (6.00–180.00)
	Daily care time (hours)	7.55±9.65	2.00 (0.50–24.00)
	Cost for care (10^4^won/month)	33.17±29.27	20.00 (5.00–150.00)
	Depression (Beck depression inventory)	10.82±9.69	8.00 (0.00–38.00)
	Caregiver burden inventory (total score)	51.95±19.93	49.00 (25.00–111.00)
	time-dependence burden	12.38±5.24	12.00 (5.00–25.00)
	developmental burden	8.87±4.36	8.00 (4.00–20.00)
	physical burden	6.19±3.14	6.00 (3.00–15.00)
	social burden	9.46±4.38	9.00 (5.00–24.00)
	emotional burden	9.41±4.27	8.00 (5.00–21.00)
	economic burden	5.63±2.74	5.00 (3.00–14.00)
	Understanding of the disease (total score)	11.92±3.64	13.00 (3.00–19.00)
	pathophysiology	3.39±1.24	3.00 (0.00–5.00)
	symptoms	3.28±1.62	3.00 (0.00–6.00)
	treatment	5.24±1.79	5.00 (2.00–8.00)

### Factors related to caregiver burden

The patient-related factors having a significant correlation with the burden of care were the gender of the patients and the K-MMSE (Table 2). Female patients and lower K-MMSE increased the burden of care. Among the caregiver-related factors, the gender of caregiver, income, duration of care, daily care time, relationship to patient, and understanding of the disease were found to be correlated with the burden of care of the primary caregivers. The burden of care was higher in cases of male caregivers, low income, long care duration, long daily care time, spouse as the primary caregiver, and low level of disease understanding, particularly in physiopathology/diagnosis.

**Table 2:** Pearson’s correlation analysis with caregiver burden inventory.

	CBI total score	time-dependency	develop-mental	physical	social	emotional	economic
**Patients’ characteristics**							
Sex	-.206*(.015)	-.120(.158)	-.159(.060)	-.245*(.004)	-.137(.106)	-.224*(.008)	-.165(.051)
Age	-.141(.097)	.031(.712)	-.179*(.034)	-.060(.481)	-.133(.118)	-.258*(.002)	-.117(.168)
education	.096(.264)	-.026(.759)	.128(.135)	.146(.088)	.057(.506)	.192*(.025)	-.011(.895)
disease duration	.154(.071)	.099(.247)	.168*(.049)	.143(.094)	.183*(.032)	.085(.321)	.075(.383)
HY stage	.057(.525)	.184*(.040)	.008(.926)	.019(.836)	.002(.980)	.008(.930)	.008(.932)
K-MMSE	-.202*(.021)	-.202*(.022)	-.153(.083)	-.154(.081)	-.188*(.033)	-.117(.186)	-.154(.082)
**Caregivers’ characteristics**							
Sex	.198*(.019)	.085(.314)	.161(.057)	.296*(.000)	.172*(.042)	.195*(.021)	.102(.230)
Age	.136(.107)	.089(.291)	.085(.319)	.119(.160)	.047(.582)	.138(.103)	.260*(.002)
education	.096(.264)	-.026(.759)	.128(.135)	.146(.088)	.057(.506)	.192*(.025)	-.011(.895)
Income	-.195*(.020)	-.148(.081)	-.142(.094)	-.183*(.030)	-.085(.314)	-.107(.207)	-.399*(.000)
Relation	-.257*(.011)	-.113(.273)	-.214*(.036)	-.167(.104)	-.123(.231)	-.346*(.001)	-.370*(.000)
care duration (m)	.191*(.024)	.137(.107)	.208*(.014)	.155(.068)	.177*(.037)	.097(.257)	.183*(.031)
daily care time (h)	.413*(.000)	.458*(.000)	.372*(.000)	.359*(.000)	.233*(.005)	.305*(.000)	.278*(.001)
care cost	.121(.153)	.161(.056)	.183*(.030)	.133(.116)	.039(.644)	.031(.713)	.017(.845)
Understanding of the disease							
total score	-.042(.646)	.017(.849)	.032(.725)	-.022(.810)	-.053(.566)	-.099(.280)	-.135(.140)
pathophysiology	-.201*(.027)	-.139(.128)	-.131(.153)	-.111(.226)	-.151(.099)	-.240*(.008)	-.263*(.004)
symptoms	.085(.355)	.118(.196)	.130(.154)	.094(.307)	.020(.830)	.038(.682)	-.013(.884)
treatment	-.028(.760)	.036(.698)	.023(.803)	-.049(.591)	-.028(.758)	-.066(.469)	-.108(.240)

* p < .05; Dummy variables: sex (female:0, male:1), relationship (spouse:0, others:1)

To determine which factors contributed most to caregiver burden, we performed a stepwise linear regression analysis, considering all significant factors that were shown to have an association with caregiver burden in univariate linear regression analysis as covariates in a multivariate stepwise regression analysis. The most important predictive factor was daily care time, followed by sex of patient, which together accounted for 25% of the variance in caregiver burden (Table 3). Daily care time was found to have a major influence on caregiver burden. In addition, caregiver burden was higher when the patient was female.

**Table 3:** Results of stepwise multivariate regression analysis of caregiver burden.

Predictors	Standardized regression coefficient	R^2^	R^2^ change	t value	p value	
Daily care time	0.362	0.177		3.289	.002	
Sex of patient	-0.274	0.249	0.072	-2.490	.015	
					multivariate	R = 0.499
						R^2^ = 0.249
					adjusted	R^2^ = 0.226

### Correlation of relationship between primary caregiver and patient to burden of care

As the burden of care was found to be dependent on the relationship between the patients and the primary caregivers, the difference depending on the caregivers' relationship with the patients was investigated [15]. Among the 96 primary caregivers actually living together with the patients, the spouses (56 subjects) were the caregivers more often than the offspring (37 subjects) (Table 4). In the spouse group, the number of female patients was more than that of male patients, and the patients were younger and had a higher level of education. The spouses were older, had a lower level of education, and had a lower income. Spouses had a longer daily care time and were more depressed than offspring. The emotional burden and the economic burden were greater and the level of understanding of the disease was lower in the spouse group.

**Table 4:** Comparison of characteristics of caregivers according to relationship with patient.

		Spouse (n = 59)	Offspring (n = 37)	p
**Patient characteristics**				
	Male : Female	27 : 32	34 : 3	< .001
	Age (years)	68.79±7.17	71.82±7.38	.024
	Hoehn and Yahr stage	2.41±0.58	2.59±0.53	.060
	Disease duration (months)	60.93±45.83	80.27±57.01	.111
	Education (years)	9.69±4.08	6.62±4.89	.004
	K-MMSE score	26.55±3.16	24.79±4.51	.095
**Caregivers’ characteristics**				
	Male : Female	32 : 27	14 : 23	.059
	Age (years)	65.89±10.72	44.26±11.47	< .001
	Education (years)	7.53±5.26	9.94±4.26	.024
	Income (10^4^won/month)	177.12±126.31	274.32±118.95	< .001
	Care duration (months)	63.25±46.75	75.08±59.17	.513
	Daily care time (hours)	12.14±10.92	5.51±8.17	.005
	Cost for care (10^4^won/month)	33.81±25.87	37.57±29.03	.654
	Depression (Beck depression inventory)	12.32±10.08	7.46±6.52	.024
	Caregiver burden inventory (total score)	58.24±21.19	47.35±18.23	.012
	time-dependence burden	13.49±5.72	12.22±5.18	.328
	developmental burden	10.10±4.68	8.11±4.11	.046
	physical burden	6.80±3.41	5.68±2.99	.115
	social burden	10.27±4.44	9.14±4.58	.174
	emotional burden	10.73±4.80	7.62±2.79	.003
	economic burden	6.85±3.15	4.59±2.05	< .001
	Understanding of the disease (total score)	10.80±3.64	12.97±2.95	.004
	Pathophysiology	2.95±1.16	3.79±1.02	.002
	Symptoms	3.05±1.67	3.42±1.46	.312
	Treatment	4.75±1.89	5.76±1.48	.015

When the caregiver was the spouse, the most important predictive factor for caregiver burden was daily care time (Table 5). Understanding the pathophysiology of PD, which is a subcategory of disease understanding, was also a significant factor affecting the caregiver burden. These two variables together accounted for 34% of the variance of the caregiver burden when the caregiver was the spouse. On the other hand, no significant factors were detected in a multivariate linear regression analysis when the caregiver was an offspring.

**Table 5:** Results of stepwise multivariate regression analysis of caregiver burden in spouse group.

Predictors	Standardized regression coefficient	R^2^	R^2^ change	t value	p value	
Daily care time	0.579	0.257		4.163	< .001	
Score of pathophysiology	-0.302	0.343	0.086	-2.172	.037	
					multivariate	R = 0.586
						R^2^ = 0.343
					adjusted	R^2^ = 0.307

## Discussion

The present study was conducted to identify factors associated with the burden of care of the PD caregivers and verify the effect of understanding the disease. The key results of the present study are described below.

The caregiver burden was increased in female patients, low cognition patients, long daily care time, care duration, male caregivers, and spouses of the patients. The findings regarding caregiver burden related to care time and economic level of the caregivers and spouses were consistent with those of previous reports, but the psychological factors that were known to be correlated, such as the depression and anxiety of the caregivers, were not verified in the present study [16,17]. While some studies showed that the caregiver burden was slightly greater or the quality of life was slightly lower in the caregivers of male patients, the results of the present study showed that the caregiver burden was higher in the cases of female patients and male caregivers [9,16,18]. Cultural difference of caregiving could be considered as a cause of increased burden in female patients and male caregivers, but the authors could not find the supporting evidence in the field of PD research. One study found gender difference in caregiving and reported that women with PD had lower resources of informal caregiving [19]. Based on such finding, it can be presumed that female patients accompanied by family member may have more disability and can increase the burden of care. Further studies are needed for the gender difference and cultural difference of caregiving PD patients. The correlation of the level of disease understanding of the caregivers, especially in pathophysiology /diagnosis, with the burden of care found in the univariate analysis, was considered an important result that may support the efficacy of disease educational programs, however no significant correlation was found in multivariate analysis. The significant factors that appeared in the multivariate analysis were daily care time and female patients. The daily care time was found to be the factor having the greatest effect, since it represented the dependence of patients on caregivers for activities of daily living caused by the disease. However, as mentioned above, additional studies need to be conducted on the association of patient gender and burden of care.

To investigate the burden of care according to the caregivers' relationship with the patients, caregivers living together with the patients were divided into spouses and offspring. The results showed that the emotional and economic burdens of care were higher in primary caregivers who were spouses of the patients. The spouses had a longer daily care time, were more depressed, earned less income, and received less education than the offspring. The level of understanding of the disease by the spouses was lower than the offspring for physiopathology/diagnosis and treatment/progress. This result was consistent with a previous report showing that spouses had a greater burden of care than offspring, and that the burden of care was highly-correlated with depression in the spouses and local social support in the offspring [15]. The present study identified daily care time as the most significant factor correlated with the burden of care in spouses. This result is consistent with previous reports, which showed daily care time effected caregiver burden [15,16].

The results of the present study also verified that disease understanding by the patient’s spouse was significantly correlated with the burden of care, which has not been investigated before. This result strongly supports the efficacy of a disease education program, and provides grounds for an educational program to be applied, not just to provide psychosocial support to the caregivers of PD patients, but also to relieve the burden of care. As previously described, the PEPP focused on the psychological factors and included contents about stress management, health promotion, and improvement of quality of life [20,21]. With the changes in the society, demographic structure, family structure, and values, the care by family members, which used to be taken for granted, has been reduced, and a novel approach is required to address the burden of care [22]. While research on the burden of PD care has been focused on the individual factors of family caregivers, future studies must analyze the social and external factors to develop methods to reduce the burden of care. The results of the present study showed that the level of disease understanding of the family caregivers correlated with the burden of care. This emphasizes the need for establishing a PD disease education program in local communities to improve the social support or external factors related to the burden of care. Though we found a correlation between understanding and burden of care, such correlation was found only in the area of pathophysiology/diagnosis. It was predicted that the understanding of the patient's various motor and non-motor symptoms, and understanding of disease progression and suitable treatment for each disease condition might be also important to the overall understanding of the disease. Further studies may need to be conducted to identify the factors that are necessary to complete this understanding in the caregivers. Education may be provided more efficiently by preparing an educational program based on the results that will be obtained from future studies. The results of the present study may provide the basis for developing an educational program for increasing the understanding about the disease to reduce the burden of care of the primary caregivers for patients with chronic neurodegenerative diseases, including PD.

The present study was conducted with limited research subjects who were patients visiting a specific hospital, together with their primary caregivers, and thus generalization of the results is limited. In particular, since the patients with a cognitive disorder, which greatly affects the burden of care, were excluded from the present study, further studies need to be conducted including those subjects. And, we did not evaluate the motor and non-motor aspects of disease (unified Parkinson’s disease rating scale and/or non-motor symptom scale) and disability in daily activity (Schwab and England activities of daily living), which could have great impacts on burden of care and should be considers as a limitation. Future study investigating full range of disease burden and daily disability is needed to verify the contribution of understanding for the burden of care in PD. The symptoms of depression were also evaluated in the present study as a psychological factor of the caregivers, but no correlation with the burden of care was found in the present study. This may be partially because of the limitations of the self-reporting questionnaire. More studies may need to be conducted using an objective inventory, including other factors, such as anxiety.

## Supporting information

S1 File Additional questions for economic burden of caregivers to caregiver burden inventory. (PDF)
