# Supplementary material for: The burden of care and the understanding of disease in Parkinson’s disease
Source: PLoS One. 2019 May 31;14(5):e0217581. doi: 10.1371/journal.pone.0217581 (PMC6544353; doi:10.1371/journal.pone.0217581)
Supplement: S1 File — (PDF) [file pone.0217581.s001.pdf]

Supporting Information

Additional questions for economic burden of caregivers to Caregiver Burden Inventory

- Original language version

아래 질문에 대해 당신에게 가장 적절한 답변을 고르시오

⓪ 전혀 아니다 ① 대체로 아니다 ② 간혹 그렇다 ③ 자주 그렇다 ④ 거의 항상 그렇다

● 내 가족은 환자에게 드는 비용 때문에 여유가 없다	⓪ ① ② ③ ④
● 환자에게 비용이 너무 많이 든다	⓪ ① ② ③ ④
● 나는 경제적인 도움을 주지 않는 친지에게 섭섭하다	⓪ ① ② ③ ④

- English version

Choose the number that best represents how often the statement describes your feelings.

⓪ Never ① Rarely ② Sometimes ③ Quite Frequently ④ Nearly Always

● My family is financially strapped due to the cost for the patient	⓪ ① ② ③ ④
● Patient costs too much	⓪ ① ② ③ ④
● I am sorry for relatives who do not provide financial help	⓪ ① ② ③ ④
